# Glial TDP-43 regulates axon wrapping, GluRIIA clustering and fly motility by autonomous and non-autonomous mechanisms

**DOI:** 10.1093/hmg/ddv330

**Published:** 2015-08-13

**Authors:** Giulia Romano, Chiara Appocher, Michele Scorzeto, Raffaella Klima, Francisco E. Baralle, Aram Megighian, Fabian Feiguin

**Affiliations:** 1 International Centre for Genetic Engineering and Biotechnology, Padriciano 99, Trieste 34149, Italy and; 2 Department of Biomedical Sciences, University of Padova, via Marzolo 3, Padova 35131, Italy

## Abstract

Alterations in the glial function of TDP-43 are becoming increasingly associated with the neurological symptoms observed in Amyotrophic Lateral Sclerosis (ALS), however, the physiological role of this protein in the glia or the mechanisms that may lead to neurodegeneration are unknown. To address these issues, we modulated the expression levels of TDP-43 in the Drosophila glia and found that the protein was required to regulate the subcellular wrapping of motoneuron axons, promote synaptic growth and the formation of glutamate receptor clusters at the neuromuscular junctions. Interestingly, we determined that the glutamate transporter EAAT1 mediated the regulatory functions of TDP-43 in the glia and demonstrated that genetic or pharmacological compensations of EAAT1 activity were sufficient to modulate glutamate receptor clustering and locomotive behaviors in flies. The data uncovers autonomous and non-autonomous functions of TDP-43 in the glia and suggests new experimentally based therapeutic strategies in ALS.

## Introduction

Amyotrophic Lateral Sclerosis (ALS) is an adult-onset disease characterized by progressive degeneration and loss of motoneurons followed by the atrophic denervation of the skeletal muscles (1). Nevertheless, the mechanisms that affect motoneurons function and generate the symptoms of the disease remain unknown. Previous studies that reported mutations in the TDP-43 gene, linked the neuronal function of this protein with the pathological manifestations of the disease (2). However, recent histological analysis revealed the presence of insoluble TDP-43 in glial tissues as well, suggesting that different cell types may also contribute to the degenerative process observed in ALS (3,4). On this basis, it has been proposed that perturbations in neuron–glia interactions could lead to neurodegeneration by different cell-autonomous and non-autonomous mechanisms (5–8). In agreement with this hypothesis, experiments performed in *Drosophila* models showed that modulations of the endogenous TDP-43 protein (TBPH) in the glia provoked locomotive defects and a reduction in life span, implying that the alterations in these tissues may affect neuronal activity and initiate the neurological symptoms of the disease (9,10). However, the physiological functions of TDP-43 in the glia or the pathological mechanisms that may affect neuronal activity remain unknown. In this manuscript, therefore, we analyze the function of TBPH in the Drosophila glia and show that is essential for axon wrapping and glutamate receptor clustering *in vivo*.

## Results

### Loss of glial TBPH provokes wrapping defects in motor axons

**Figure 1.**
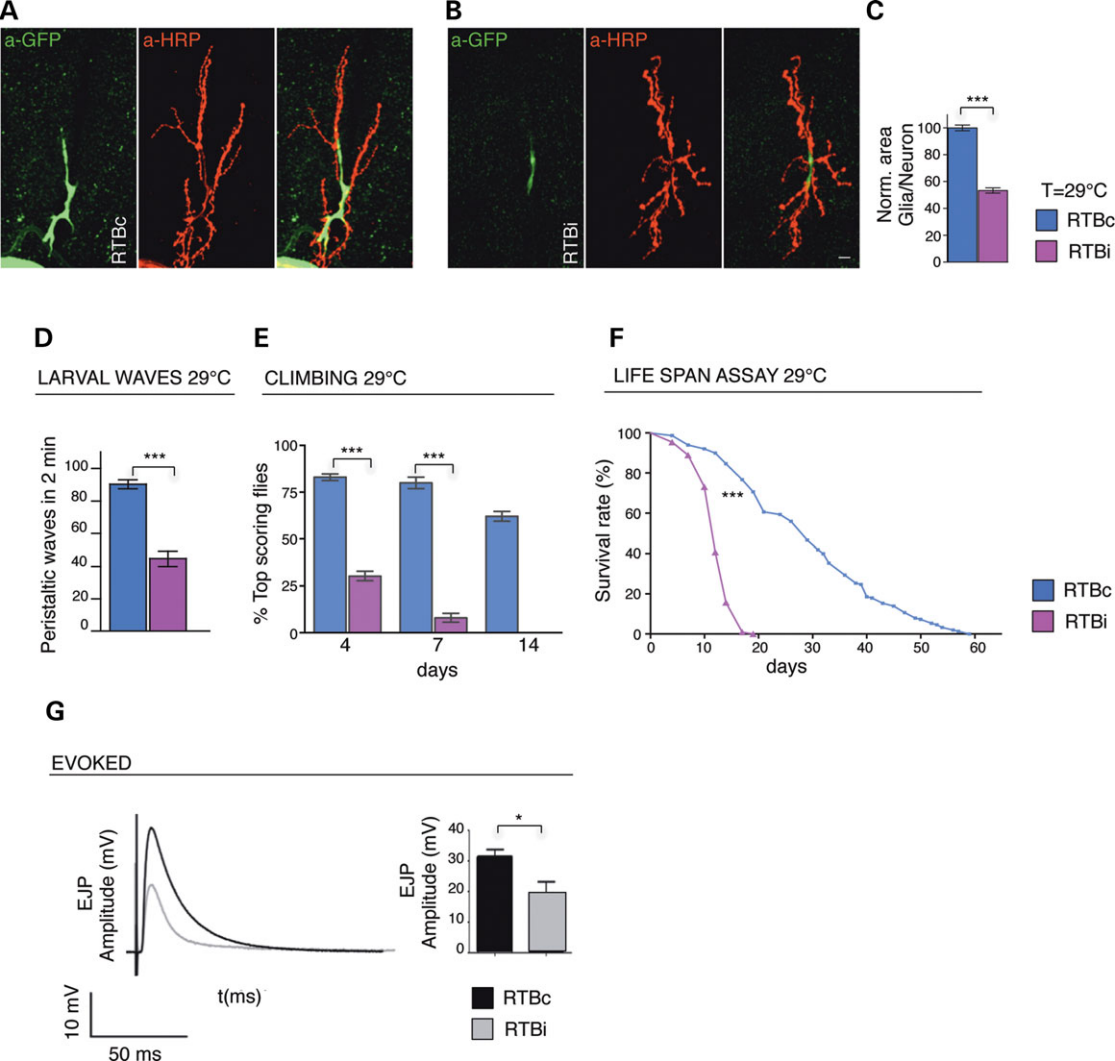
Silencing of TBPH in glia provokes reduction in the wrapping glia, functional and locomotive defects. (**A,B**) Confocal images of third instar larvae (L3), NMJs expressing mCD8-GFP using *repo*-GAL4 double labeled with anti-GFP (in green) and anti-HRP (in red) in (**A**) RTBc (UAS-Dcr2;*tbph*^Δ*23*^/+;*repo*-GAL4,UAS-GFP/+) and in (**B**) RTBi (UAS-Dcr2;*tbph*^Δ*23*^/+;*repo*-GAL4,UAS-GFP/TBPH-RNAi). (**C**) Quantification of glial area, *n* = 15 larvae. (**D**) Number of peristaltic waves of RTBc and RTBi larvae. *n* = 30. (**E**) Climbing assay at days 4, 7 and 14 in RTBc and RTBi adult flies, *n* = 100. (**F**) Analysis of life span in RTBc and RTBi adult flies, *n* = 100. (**G**) Evoked neurotransmitter release. Left panel: Representative EJPs evoked by segmental nerve stimulation in fiber 6/7 of A3 of RTBc (black trace) and RTBi (gray trace) in third instar larvae. Calibration on the left side. Right panel: Mean Amplitude (± SEM) of Excitatory Post Synaptic Potentials (EJPs) evoked by segmental nerve stimulation and recorded in muscle 6/7 of segment A3/4 of third instar larvae, RTBc (n = 6,12) and RTBi (n = 5,10). *n* = number of larvae and number of fibers analyzed, respectively. For each fiber 15 EPPs following 0.5 Hz stimulation were considered. ***P < 0.001 *P < 0.05, using T-test, log-rank test (life span). Scale bar 10 µm. Error bars SEM.

In agreement with previous reports, we confirmed that the TBPH protein co-localized with the reversed polarity protein (Repo) in the nucleus of the glial cells present in different regions of Drosophila brain (Supplementary Material, Fig. S1A–D) and observed that the expression of RNA interference (RNAi) against TBPH in these tissues, using repo-GAL4 (Supplementary Material, Fig. S2A,B), provoked strong motility defects as well as the consistent reduction of the life span (Fig. 1D–F). To understand the molecular mechanisms behind these locomotive phenotypes, we decided to analyze whether the reduction of TBPH in the glia affected the functional organization of the neuromuscular junctions (NMJ). Therefore, we observed that the peripheral glia almost entirely wraps the surface of the motoneurons axons present at the NMJ in mature L3 larvae by extending their cytoplasm along the presynaptic branches (Fig. 1A). Although we concentrated the analysis on muscles 6 and 7, we found that in almost every neuromuscular synapsis the glial membranes form gliopod-like projections and lamellipodia-like expansions that engage a consistent surface of the NMJ area (11). On the other hand, we found that the expression of TBPH-RNAi with *repo-*GAL4 induced a strong reduction in the neuromuscular area covered by the glia compared with controls (Fig. 1B,C). Intriguingly, we observed that these phenotypes were accompanied by a significant reduction in the evoked junction potentials (EJPs) registered in TBPH silenced glia compared with controls (Fig. 1G), indicating that the synaptic transmission was altered in the NMJs. On the contrary, we found that the glial suppression of TBPH did not affect the number of synaptic boutons or terminal branches formed at the NMJs and did not provoked motoneurons defects in the synaptic vesicle exocytosis process as quantified with the FM1-43 dye in loading/unloading *in vivo* assay (see Supplementary Material, Fig. S2E,F), implying that the locomotive and neurological problems described above, may not derive from primary defects in motoneurons development (Fig. 1A,B a-HRP panel, quantified in Supplementary Material, Fig. S2C,D).

**Figure 2.**
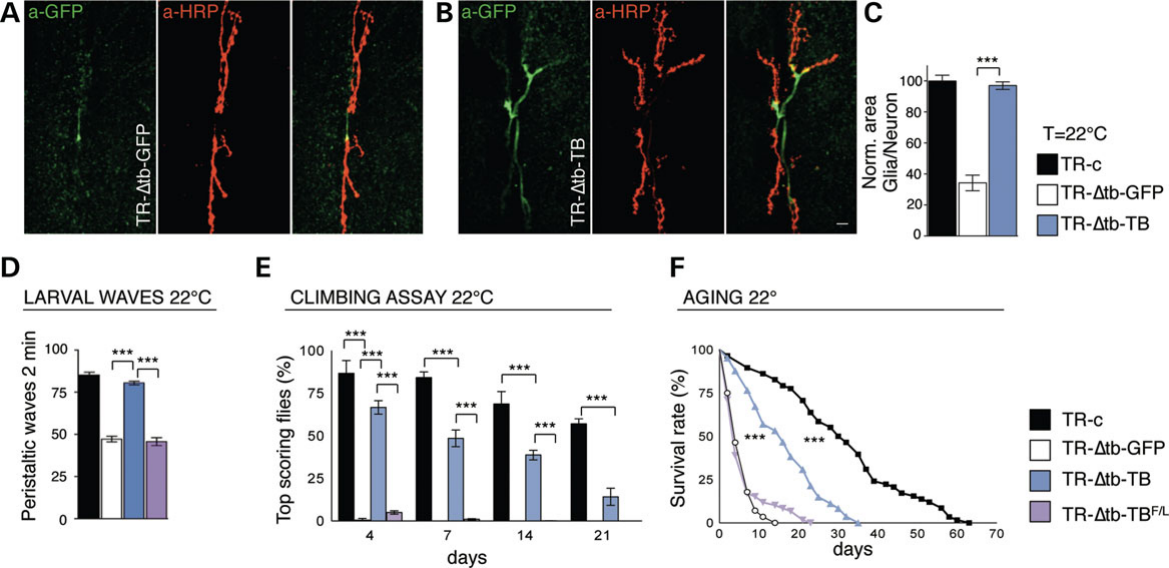
The constitutive expression of TBPH in glia restores the wrapping defects, the locomotive impairments and life span of TBPH minus flies. (**A,B**) Confocal images of third instar larvae (L3) NMJs expressing mCD8-GFP using *repo*-GAL4 double labeled with anti-GFP (in green) and anti-HRP (in red) in (**A**) TR-Δtb-GFP (*tubulin*-GAL80^TS^,*tbph*^Δ*23*^/*tbph*^Δ*23*^;*repo*-GAL4,UAS-GFP/+) and in (**B**) TR-Δtb-TB (*tubulin*-GAL80^TS^,*tbph*^Δ*23*^*/tbph*^Δ*23*^,UAS-TBPH;*repo*-GAL4,UAS-GFP/+). (**C**) Quantification of glial area, *n* = 15 larvae. (**D**) Number of peristaltic waves of TR-c (*tubulin*-GAL80^TS^,*tbph*^Δ*23*^/+;*repo*-GAL4,UAS-GFP/+), TR-Δtb-GFP, TR-Δtb-TB and TR-Δtb-TB^F/L^ (*tubulin*-GAL80^TS^,*tbph*^Δ*23*^/*tbph*^Δ*23*^;*repo*-GAL4,UAS-GFP/UAS-TBPH^F/L^) larvae, *n* = 30. (**E**) Climbing assay on adult flies at days 4, 7, 14 and 21, *n* = 100. (**F**) Aging assay on adult flies, *n* = 100. ***P < 0.001 calculated by one-way ANOVA, log-rank test (life span). Scale bar 10 µm. Error bars SEM.

Further support to these findings came from the characterization of the cytoplasmic area covered by peripheral glia in TBPH null flies, that appeared strongly reduced, compared with wild-type controls (Fig. 2A–C). Interestingly, we found that the glial expression of the endogenous protein was able to significantly rescue the morphological defects in the glial shape, the alterations in locomotive behaviors and the life span reduction described in TBPH null backgrounds from larval stages to adulthood (Fig. 2B-F and Supplementary Material, Fig. S4A–C) (12). Similar genetic rescue was obtained by expressing the human TDP-43 protein in the Drosophila glia (Supplementary Material, Fig. S3). On the contrary, flies expressing the RNA-binding defective version of TBPH (TBPH^F/L^) were not able to recover the TBPH mutant phenotypes (Fig. 2D–F) (13). These data demonstrated that the glial function of this protein is conserved and proposed that analogous alterations in TDP-43 activity, may autonomously initiate or maintain the pathological processes in patients (14–16).

### The glial role of TBPH promotes synaptic growth and glutamate receptor clustering

**Figure 3.**
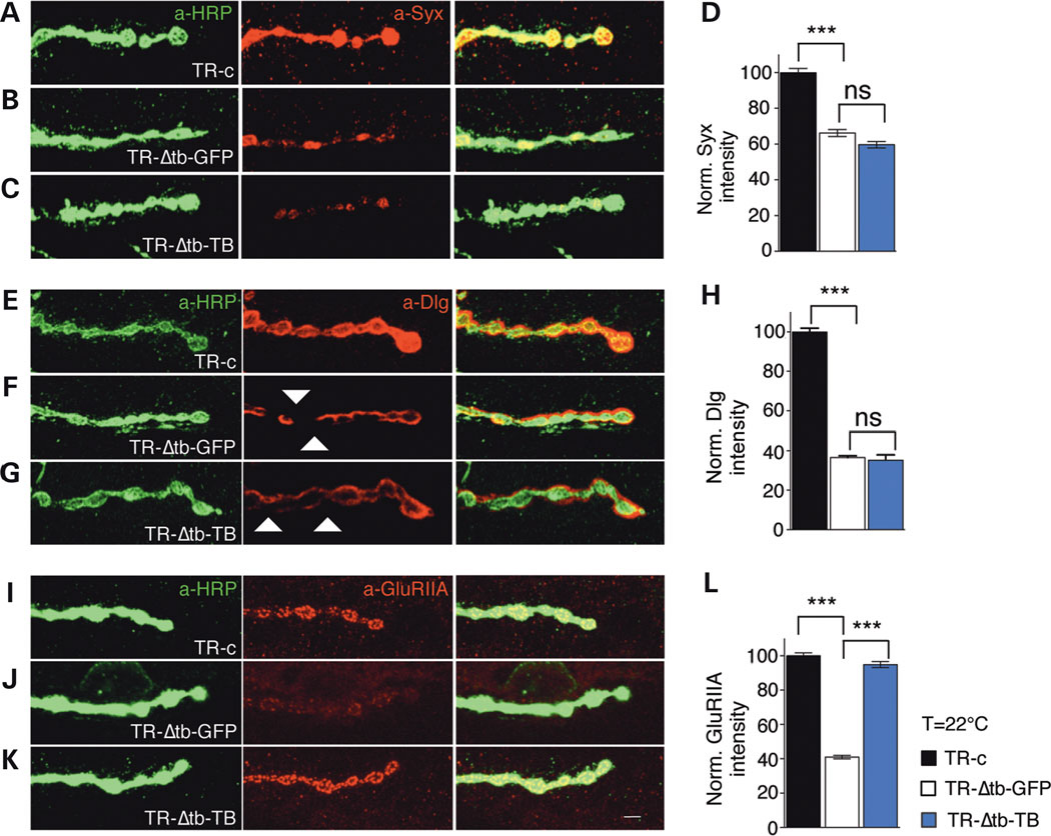
TBPH function in the glia is required to promote GluRIIA clusters formation at the postsynaptic membranes. (**A**–**C**) Confocal images of L3 NMJ terminals in muscle 6/7 stained with anti-HRP (in green) and anti-Syx (in red) in (**A**) TR-c (*tubulin*-GAL80^TS^,*tbph*^Δ*23*^/+;*repo*-GAL4,UAS-GFP/+), in (**B**) TR-Δtb-GFP (*tubulin*-GAL80^TS^,*tbph*^Δ*23*^/*tbph*^Δ*23*^;*repo*-GAL4,UAS-GFP/+) and in (**C**) TR-Δtb-TB (*tubulin*-GAL80^TS^,tbph^Δ23^*/tbph*^Δ*23*^,UAS-TBPH;*repo*-GAL4,UAS-GFP/+). (**D**) Quantification of Syx intensity, *n* = 200 boutons. (**E**–**G**) Confocal images of L3 NMJ terminals in muscle 6/7 stained with anti-HRP (in green) and anti-Dlg (in red) in TR-c, TR-Δtb-GFP and TR-Δtb-TB. (**H**) Quantification of Dlg intensity, *n* = 200 boutons. (**I–K**) Confocal images of L3 NMJ terminals in muscle 6/7 stained with anti-HRP (in green) and anti-GluRIIA (in red) in TR-c, TR-Δtb-GFP and TR-Δtb-TB. (**L**) Quantification of GluRIIA intensity, *n* = 200 boutons, ns = not significant, ***P < 0.001 calculated by one-way ANOVA. Scale bar 5 µm. Error bars SEM.

A deeper analysis of *tbph*^Δ*23*/−^ NMJs, rescued with *repo-*GAL4 showed that the glial expression of TBPH stimulated the non-autonomous growth of the motoneuron axons and the formation of new synaptic terminals (Fig. 2A,B a-HRP panel, quantified in Supplementary Material, Fig. S4H). However, the final morphology of the newly formed boutons appeared slightly impaired with terminal axons irregularly shaped (Supplementary Material, Fig. S4D–G). At the molecular level, we found that the intensity levels of the vesicular presynaptic protein Syntaxin (Syx) were not rescued by the expression of TBPH in the glia (Fig. 3A–D). In consonance with these presynaptic defects, we found that the postsynaptic distribution of the protein Disc-large (Dlg) was also not recovered, indicating that the neurotrophic function promoted by TBPH in the glia was not sufficient to completely differentiate these structures (Fig. 3E–H). On the opposite, we found that the glutamate receptors (GluRIIA) consistently recovered their characteristic distribution in well-defined ring-shaped clusters at the postsynaptic membranes (Fig. 3I–L), implying that the glial function of TBPH was able to influence the organization of the GluRIIA at the NMJs.

### The formation of GluRIIA clusters is early affected by TBPH dysfunctions in glia

**Figure 4.**
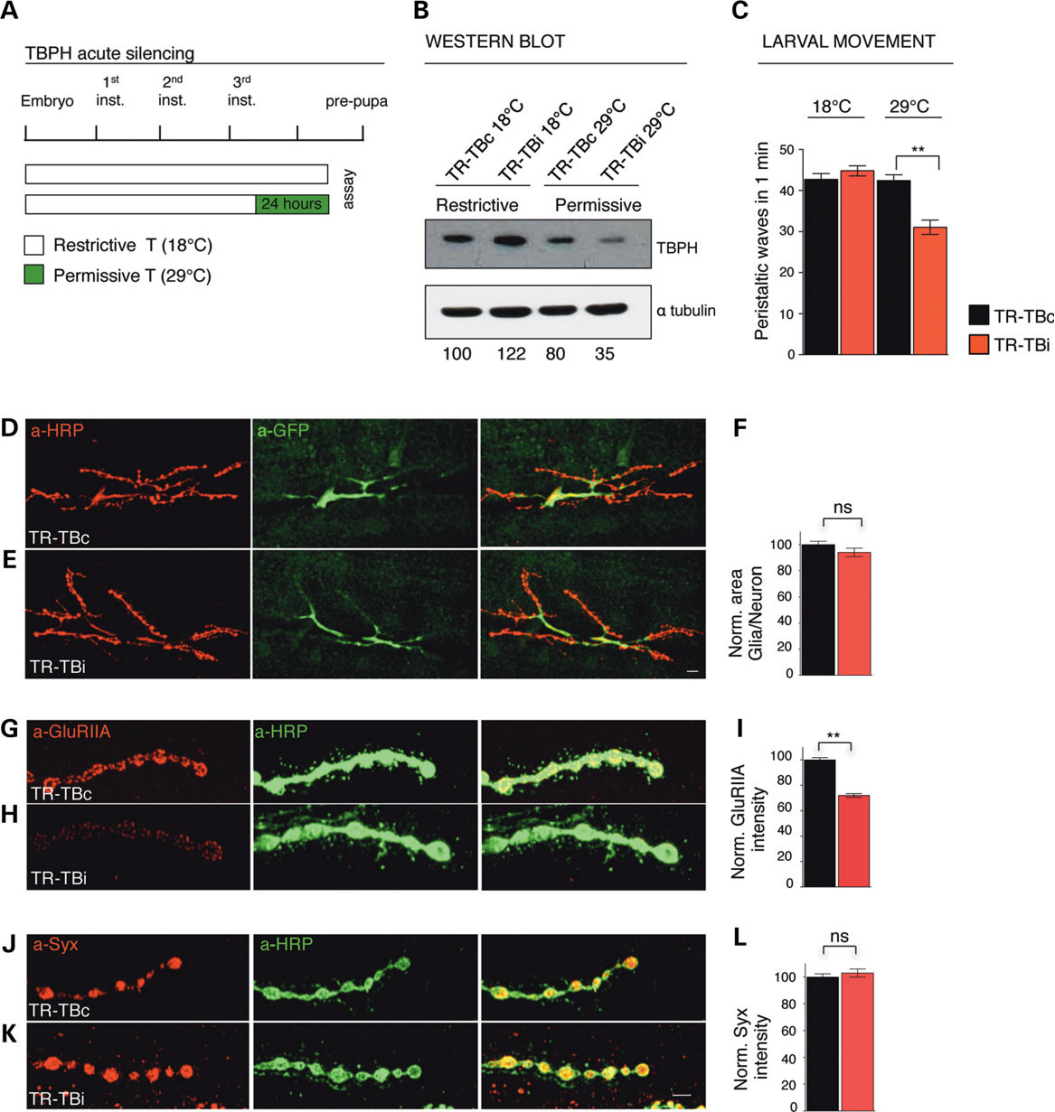
Acute silencing of glial TBPH in mature larvae. (**A**) Schematic time line of TBPH silencing in L3 larvae through TARGET system. (**B**) Western blot analysis on larval brains probed for TBPH and alpha-tubulin in TR-TBc (UAS-Dcr2;*tubulin*-GAL80^TS^,*tbph*^Δ*23*^/+;*repo*-GAL4,UAS-GFP/+) and TR-TBi (UAS-Dcr2;*tubulin*-GAL80^TS^,*tbph*^Δ*23*^/+;*repo*-GAL4,UAS-GFP/TBPH-RNAi), using the TARGET system to activate RNAi expression (24 h at 29°C). Quantification of normalized protein amount was reported below each lane, *n* = 3. (**C**) Number of peristaltic waves in TR-TBc and TR-TBi L3 larvae, *n* = 30. (**D,E**) Confocal images of third instar NMJs expressing mCD8-GFP in glial cells using repo-GAL4 double labeled with anti-HRP (in red) and anti-GFP (in green) in (**D**) TR-TBc and in (**E**) TR-TBi. (**F**) Quantification of glial area, *n* = 15. (**G,H**) Confocal images of third instar NMJs double labeled with anti-HRP (in green) and anti-GluRIIA (in red) in (**G**) TR-TBc and in (**H**) TR-TBi. (**I**) Quantification of GluRIIA intensity. (**J,K**) Confocal images of third instar NMJs double labeled with anti-HRP (in green) and anti-Syx (in red) in (**J**) TR-TBc and in (**K**) TR-TBi. (**L**) Quantification of Syx intensity. *n* = 200 boutons. Scale bar 10 µm (D,E) and 5 µm (G,H,J,K). ns = not significant, **P < 0.01 calculated by one-way ANOVA, T-test. Error bars SEM.

**Figure 5.**
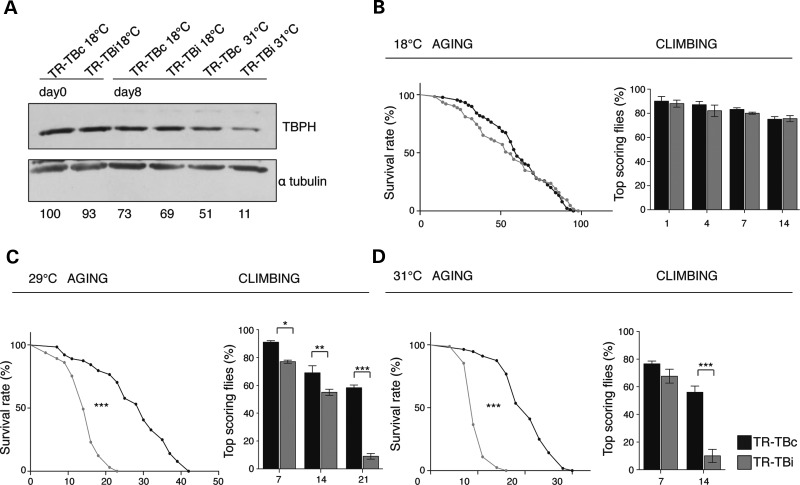
Acute silencing of glial TBPH in adult flies. (**A**) Western blot analysis of *Drosophila* adult heads, probed with anti-TBPH and alpha-tubulin before and after 8 days of TARGET mediated TBPH-RNAi expression in TR-TBc (UAS-Dcr2; *tubulin*-GAL80^TS^,*tbph*^Δ*23*^/+;*repo*-GAL4,UAS-GFP/+) and in TR-TBi (UAS-Dcr2; *tubulin*-GAL80^TS^,*tbph*^Δ*23*^/+;*repo*-GAL4,UAS-GFP/TBPH-RNAi) adult flies. Quantification of normalized protein amount was reported below each lane, *n* = 3. (**B**) Life span and climbing assay performed in TR-TBc and TR-TBi adult flies, maintained at restrictive temperature (18°C). (**C,D**) Life span and climbing assay performed in TR-TBc and TR-TBi adult flies shifted at two different permissive temperatures, (**C**) 29°C and (**D**) 31°C, *n* = 200, *P < 0.05, **P < 0.01, ***P < 0.001 calculated by one-way ANOVA, log-rank test. Error bars SEM.

In order to address whether the distribution and clustering of the GluRIIA were directly modified by the function of TBPH in glia, we blocked exclusively for 24 h TBPH expression in the glia of mature L3 larvae, using the TARGET system, as schematically reported in (Fig. 4A). We found that the acute suppression of TBPH (Fig. 4B) induced severe locomotive problems in RNAi treated larvae compared with controls (Fig. 4C) but not related modifications in the glial shape (Fig. 4D–F). Similar motility defects, followed by premature death, were obtained after the acute silencing of the TBPH protein in adult flies (Fig. 5A–D), proving the continuous necessity of TBPH function in these tissues during the Drosophila life cycle. Interestingly, we observed that the acute TBPH suppression provoked immediate modifications in the GluRIIA distribution and a significant reduction in the intensity levels of the receptor clusters present at the postsynaptic membranes (Fig. 4G–I). These defects became more evident after longer RNAi treatments performed during larval development (Supplementary Material, Fig. S5D–F), suggesting that TBPH is permanently required in the glia to maintain the postsynaptic distribution of these structures. In contrast, no modifications were appreciated in the presynaptic levels of Syx (Fig. 4J–L) and no defects were detected either in the postsynaptic distribution of Dlg (Supplementary Material, Fig. S5A–C) or in the anatomy of the presynaptic terminals (Supplementary Material, Fig. S2C–D). These results indicated that early defects in the glial function of TBPH differentially influenced these structures and, moreover, suggested tissue-specific regulatory functions of TBPH in the glia in addition to neurons (17).

**Figure 6.**
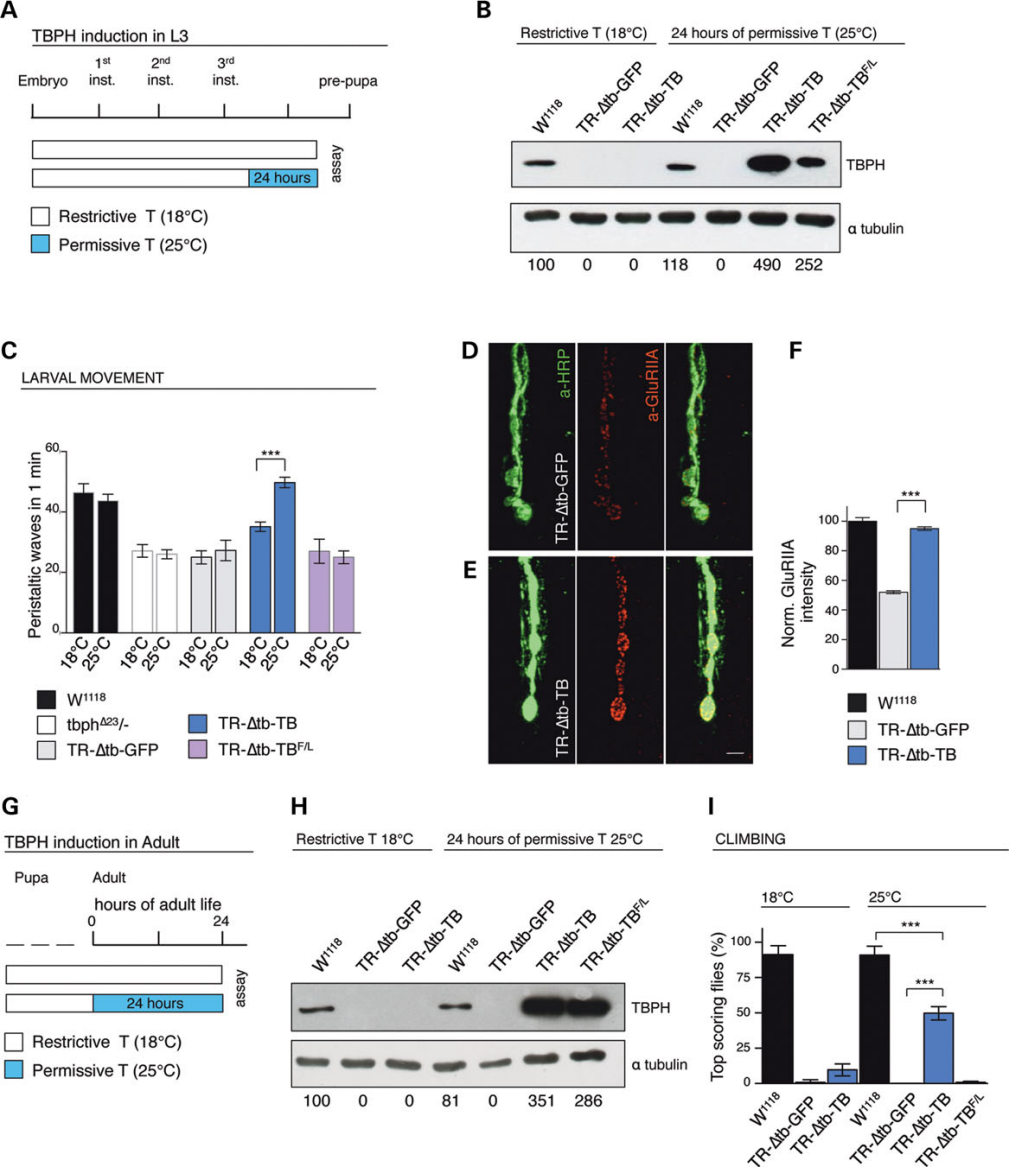
Late expression of TBPH transgene in glia recovers mutant phenotype defects. (**A**) Schematic time line of TBPH induction in L3 larvae through TARGET system. (**B**) Western blot of L3 brains probed with anti-TBPH and alpha-tubulin before and after 24 h of TARGET mediated transgene expression in W^1118^, TR-Δtb-GFP (*tubulin*-GAL80^TS^,*tbph*^Δ*23*^/*tbph*^Δ*23*^;*repo*-GAL4,UAS-GFP/+), TR-Δtb-TB (*tubulin*-GAL80^TS^,tbph^Δ23^*/tbph*^Δ*23*^,UAS-TBPH;*repo*-GAL4,UAS-GFP/+) and TR-Δtb-TB^F/L^ (*tubulin*-GAL80^TS^,*tbph*^Δ*23*^/*tbph*^Δ*23*^;*repo*-GAL4,UAS-GFP/UAS-TBPH^F/L^). Quantification of normalized protein amount was reported below each lane, *n* = 3. (**C**) Number of peristaltic waves of W^1118^, *tbph*^Δ*23/−*^*,* TR-Δtb-GFP, TR-Δtb-TB and TR-Δtb-TB^F/L^ larvae, *n* = 20. (**D,E**) Confocal images of L3 NMJ terminals in muscle 6/7 stained with anti-HRP (in green) and anti-GluRIIA (in red) in (**D**) TR-Δtb-GFP and (**E**) TR-Δtb-TB. (**F**) Quantification of GluRIIA intensity, *n* = 200 boutons. (**G**) Schematic time line of TBPH induction in adult flies through TARGET system. (**H**) Western blot of adult heads probed for anti-TBPH and alpha-tubulin before TBPH induction at restrictive temperature (18°C) and after 24 h of TBPH induction through shift at permissive temperature (25°C). Quantification of normalized protein amount was reported below each lane, *n* = 3. (**I**) Climbing assay of adult flies before and after 24 h of TARGET mediated transgene expression, *n* = 100. ns = not significant, **P < 0.01, ***P < 0.001 calculated by one-way ANOVA, T-test. Scale bar 5 µm. Error bars SEM.

Complementary experiments using TARGET system (Fig. 6A,B) indicated that the late expression of TBPH in mature *tbph*^Δ*23/−*^ glia was sufficient to regenerate the postsynaptic organization of the GluRIIA clusters (Fig. 6D–F) and rescue larval motility (Fig. 6C). Identical genetic manipulations performed in adult flies recovered the climbing abilities of these insects (Fig. 6G–I), uncovering the unexpected capacity of the glial tissues to regenerate their functions and indicating that these defects were not permanent and can be restore.

### dEAAT1 mediates GluRIIA clustering

**Figure 7.**
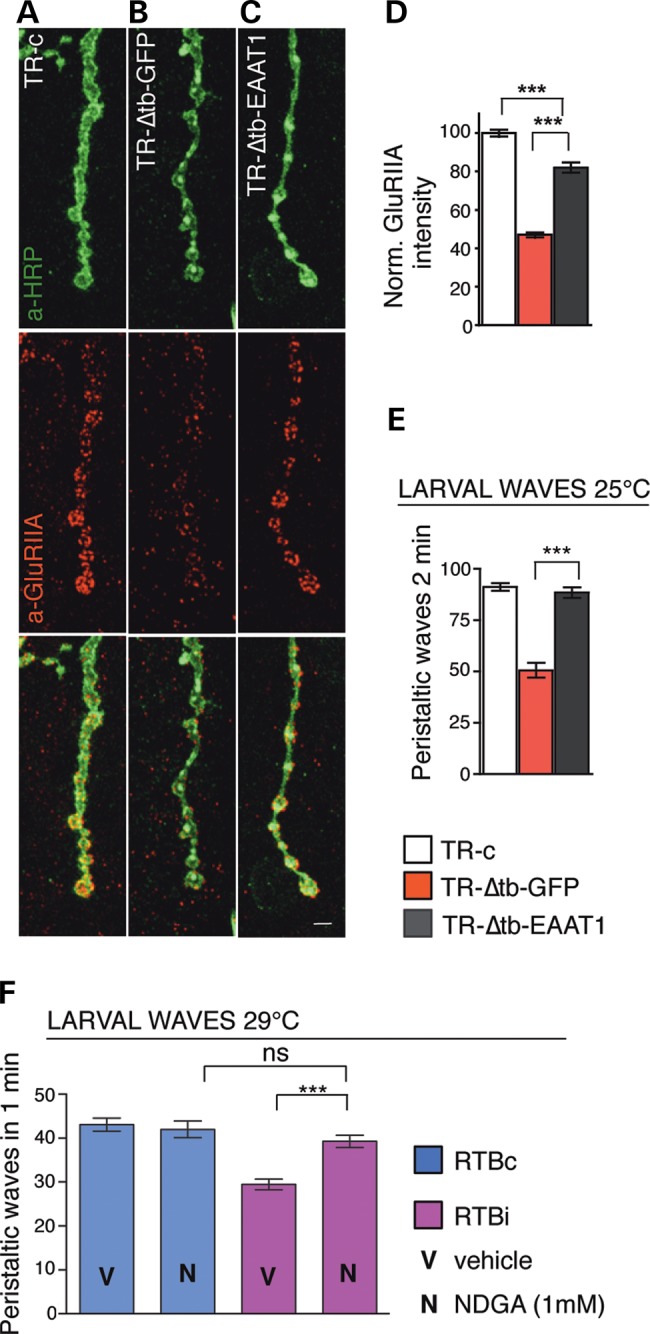
Glial dEAAT1 and NDGA mediate the defects of *tbph*^Δ*23/−*^ in GluRIIA clustering and in locomotion. (**A–C**) Confocal images of L3 NMJ terminals double labeled with anti-HRP (in green) and anti-GluRIIA (in red) in (**A**) TR-c (*tubulin*-GAL80^TS^,*tbph*^Δ*23*^/+;*repo*-GAL4,UAS-GFP/+), in (**B**) TR-Δtb-GFP (*tubulin*-GAL80^TS^,*tbph*^Δ*23*^/*tbph*^Δ*23*^;*repo*-GAL4,UAS-GFP/+) and in (**C**) TR-Δtb-EAAT1 (*tubulin*-GAL80^TS^,*tbph*^Δ*23*^/*tbph*^Δ*23*^;*repo*-GAL4,UAS-GFP/UAS-EAAT1). (**D**) Quantification of GluRIIA intensity, *n* = 200 boutons. (**E**) Number of peristaltic waves in L3 larvae, *n* = 20. (**F**) Number of peristaltic waves in L3 larvae fed in food containing 1 mm NDGA (N) or containing vehicle only (V), in RTBc (UAS-Dcr2;*tbph*^Δ*23*^/+;*repo*-GAL4,UAS-GFP/+) and RTBi (UAS-Dcr2;*tbph*^Δ*23*^/+;*repo*-GAL4,UAS-GFP/TBPH-RNAi) larvae, *n* = 20. ns = not significant, ***P < 0.001 calculated by one-way ANOVA. Scale bar 5 µm. Error bars SEM.

Defects in the glial function of TBPH seemed to affect the mRNA levels of the conserved glutamate transporters EAAT1 and EAAT2 (9) and, alterations in the extracellular levels of glutamate, apparently influence the postsynaptic GluRIIA clustering (18). In order to test these correlations, we expressed EAAT1 in the Drosophila glia and found that this protein managed to rescue the motility defects observed in TBPH minus L3 larvae (Fig. 7E). Moreover, we appreciated that EAAT1 was able to significantly recover the postsynaptic distribution and intensity levels of the GluRIIA clusters similarly to a wild-type condition (Fig. 7A–D). Instead, we detected that the glial expression of EAAT1 in TBPH minus flies was not able to rescue the defects observed in the shape of the glia or in the number of terminal branches present at the NMJs (Supplementary Material, Fig. S6A–E), indicating that different molecules may mediate the neurotrophic functions of TBPH in the glia. On the basis of these findings, we speculated that pharmacological approaches aimed to mimic the activity of EAAT1 in the clearance of glutamate might be beneficial in recovering flies motility. To evaluate this possibility, we treated Drosophila expressing TBPH-RNAi in the glia (RTBi) with the glutamate uptake enhancer nordihydroguaiaretic acid (NDGA) during Drosophila development (19). Remarkably, we observed that 1 mm of NDGA was able to recover the locomotive activity of RTBi L3 larvae to equivalent control levels (Fig. 7F), suggesting that uncontrolled levels of extracellular glutamate may cause neurotoxicity in TBPH minus flies and revealing that pharmacological actions aimed to improve the regulation of the glutamate uptake in the synaptic cleft may contribute to improve the symptoms observed in pathological conditions.

## Discussion

In addition to neurons, histological aberrations in the distribution of TDP-43 were also observed in glial cells suggesting that these tissues might be associated with the neurodegenerative process present in ALS (3,4,20). In coincidence with this idea, we found that the suppression of TDP-43 in the Drosophila glia provoked serious locomotive defects with functional alterations in synaptic transmission followed by early neurodegeneration and reduced life span but, without affecting the number or the survival rate of glial cells *in vivo* (Supplementary Material, Fig. S1E–G) (9). More importantly, our results showed that the acute reduction of TBPH function in the glia of adult flies was sufficient to initiate the typical locomotive problems observed in the disease, demonstrating that TBPH function is permanently required in these tissues to prevent neurodegeneration. Although, the Drosophila glia presents consistent molecular and cytological differences with the mammalian astrocytes, the functional characteristics of these tissues are well conserved (21). In agreement with these observations, we found that the expression of the human TDP-43 in the Drosophila glia was sufficient to rescue the motility defects observed in TBPH minus larvae, revealing that the molecular role of this protein is well-preserved and analogous consequences could be expected in TDP-43 affected patients.

### The autonomous function of TBPH is required to regulate glial shape and the subcellular wrapping of the presynaptic axons

At the subcellular level, our studies indicated that the suppression of TBPH provoked evident defects in the anatomical organization of the peripheral glia with morphological alterations in the formation of the cytosolic projections that cover the synaptic surface of the presynaptic terminal axons that constitute the NMJs (11,22). Although, we still do not know how these modifications may lead to neurodegeneration the non-autonomous defects in the transmission of the evoked potentials described in presynaptic motoneurons coincides with analogous alterations found in different experimental models as well as in patients suffering of ALS, proposing that comparable modifications may autonomously initiate the neurological symptoms of the disease or induce the pathological mechanisms of neurodegeneration (16,23).

### The glial function of TBPH regulates the postsynaptic organization of the glutamate receptors at the NMJ

**Figure 8.**
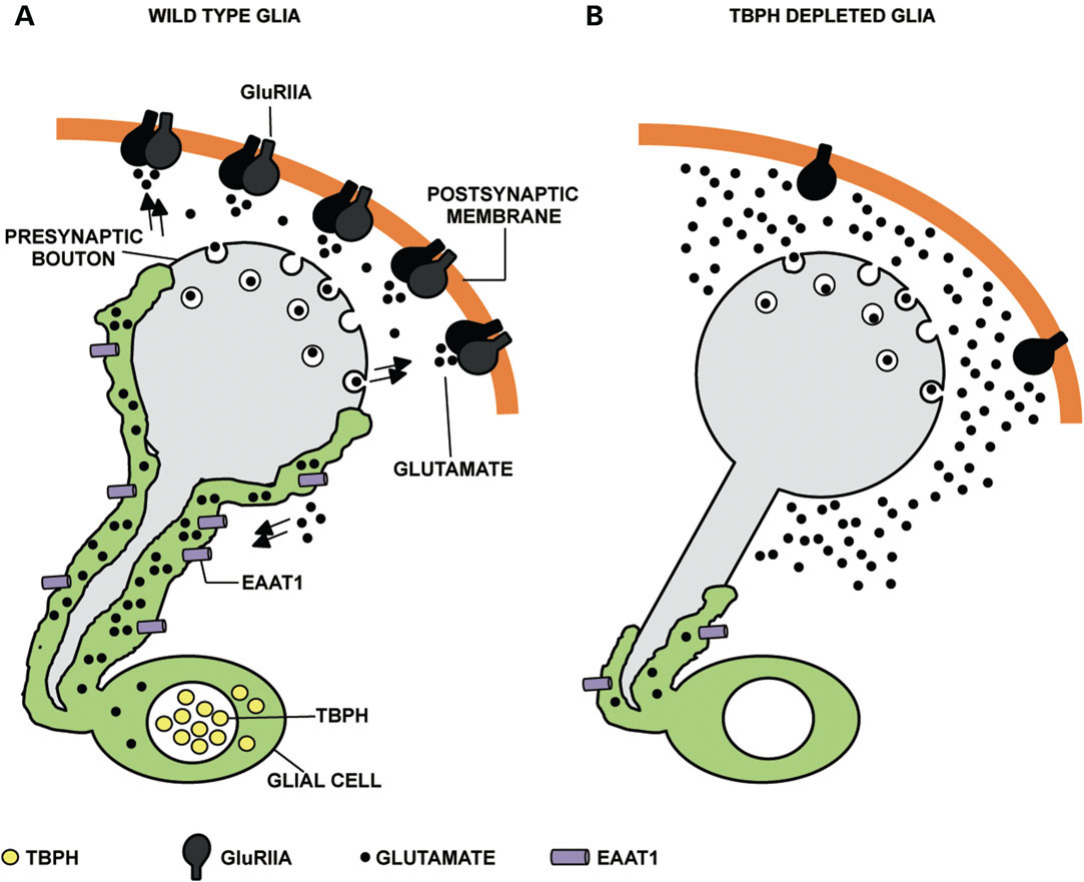
The glial function of TBPH regulates motoneurons axonal wrapping and the postsynaptic organization of the glutamate receptors clusters in the neuromuscular synapses. (**A**) In wild-type Drosophila neuromuscular synapses the peripheral glia (in green) almost completely envelops the presynaptic motoneurons axons junction (in gray) while the synaptic transmission is ensured by the vesicular release of the glutamate neurotransmitter (black dots) from the presynaptic boutons. The glutamate released in the synaptic cleft binds and induce the clustering of the glutamate receptors (GluRIIA in black) present at the postsynaptic membranes. The excess of the released glutamate is recovered through re-uptake by the EAAT1 transporter (in violet) mainly localized in the glia. (**B**) The depletion of TBPH in the glia induces the cytoplasmic retraction of these tissues, the downregulation of the EAAT1 transporter and the disruption of the GluRIIA postsynaptic clusters without affecting the presynaptic cycles of vesicles release. The model suggests that the neurotransmission defects detected in these flies might be, most probably, occasioned by the excessive accumulation of the neurotransmitter in the synaptic cleft due to the reduced capacity of the glia to uptake the glutamate.

Besides the phenotypes described above, our experiments revealed that the glial function of TBPH was sufficient to rescue the molecular levels and the wild-type distribution of the GluRIIA clusters at the postsynaptic terminals of TBPH null L3 larvae. These molecular parameters were recovered together with the locomotive abilities both in larvae and adult flies, revealing that the glial function of TBPH may directly influence these genetic traits. In agreement with this view, we found that the suppression of TBPH in the glia altered the formation of evoked synaptic potentials at the NMJ, without affecting the cycle of vesicle release in the presynaptic membranes. On the contrary, the anatomical distribution of the GluRIIA clusters present at the postsynaptic membranes was highly disturbed in the TBPH depleted glia, suggesting that electrophysiological problems originate from defects in the organization of the postsynaptic membranes (Fig. 8). The genetic rescue experiments instead, revealed that the glial function of TBPH was necessary to promote the synaptic growth of the motoneurons terminal axons during larval development. This neurotrophic function of TBPH, nevertheless, was not sufficient to rescue the neuronal levels of the vesicular protein Syx. In addition we found that the distribution of the postsynaptic protein Dlg, whose localization largely depends on the presynaptic activity of the motoneurons, was not recovered after the activation of TBPH in the glia compared with identical genetic rescue experiments performed expressing TBPH in neurons (17), revealing tissue-specific differences in TBPH function and regulatory mechanisms. Finally, the genetic rescues generated through the late expression of TBPH in mature glia indicated that the pathological defects described in locomotive behaviors and postsynaptic distribution of the GluRIIA clusters were not permanent and could be regenerated.

### Modulation of the glutamate transport in the glia suppress the pathological phenotypes described in TBPH minus flies

Defects in the regulation of the intracellular levels of the glutamate transporter EAAT1 in the glia has been associated with several neurodegenerative diseases comprising ALS. Indeed, molecular data previously generated showed that EAAT1 and EAAT2 mRNA levels are largely modified in ALS patients and, moreover, these modifications were associated with the increased oxidative stress present in the affected cases (24,25). Recent studies performed in Drosophila have also identified that EAAT1 and EAAT2 messengers were modified in TBPH minus flies suggesting that these modifications may play a role in the mechanisms behind these phenotypes despite experimental evidences were not available (9). In this manuscript, we directly tested this hypothesis and uncover that EAAT1 had an important role in the phenotypic mechanisms derived from the loss of TBPH function in the glia. The data also strongly suggest that the levels of the extracellular glutamate might be affected in TBPH null flies and responsible for the alterations in the localization of the GluRIIA at the postsynaptic membranes. These evidences, allowed to hypothesize that the implementation of pharmacological treatments aimed to enhance the glutamate uptake or counteract the oxidative stress, produced by defects in its intracellular transport, may contribute to improve the symptoms observed in TBPH minus flies (19,26). In this direction, we determined that NDGA significantly improved the locomotive capacities of TBPH-RNAi treated larvae, demonstrating the role of this protein in the organization of the NMJs and proposing that analogous situations could be expected in human pathologies associated with TDP-43 dysfunctions like ALS or FTLD.

## Material and Methods

### Fly strains

The complete genotype of the fly stocks are indicated below:

W^1118^, w; tbph^Δ23^/CyOGFP, w;;repo-GAL4, w;tubulin-GAL80^TS^, UAS-Dcr-2, w;UAS-TBPH; w;;UAS-TBPH^F/L^, w;;UAS-hTDP-43, yw;;UAS-mCD8::GFP, w;;UAS-TBPH-RNAi (ID38379, VDRC) and w;;UAS-EAAT1 (#8202, Bloomington). All TDP-43 constructs were Flag tagged and *Eco*RI-*Xba*I cloned in the pUASTattB plasmid (5xUAS, GenBank: EF362409). Transgenic flies were generated by standard embryo injection (Best Gene Inc.) through PhiC31 integrase-mediated transgenic system (27).

### Fly maintenance

All flies stocks were maintained at 25°C on a 12:12 h light:dark cycle at constant humidity on a standard cornmeal medium (agar 6 g/l, 62.5 g/l yeast, 41.6 g/l sugar, 29 g/l flour, propionic acid 4.1 ml/l). The experiments for the constitutive rescue of TBPH in glia were settled at 22°C, while the constitutive rescue of EAAT1 was reared at 25°C. All experiments related to constitutive TBPH silencing were done at 29°C.

### Larval movement

To assess the peristaltic waves of third instar larvae, we followed the previously established protocol (12). In brief, third instar larvae were selected and washed with water to remove food residues and then the larvae were transferred in a Petri dish (94 × 16) filled with a layer of 0.7% agarose in distilled water. After 30–60 s of adaptation, the larvae were analyzed by counting the number of peristaltic waves during a time span of 2 min or of 1 min. The experiments for the constitutive rescue of TBPH in glia were reared at 22°C, the constitutive rescue of EAAT1 at 25°C and the constitutive TBPH silencing at 29°C.

### Climbing assay

To assess the negative geotaxis movement in adult flies, we followed the previously established protocol (12). Shortly, groups of 20 aged flies were transferred to the bottom of a 50 ml cylinder without anesthesia. After 30 s of adaptation, climbing ability was measured by counting the flies that reached the top of the cylinder (10 cm) in 15 s. The experiments for the constitutive rescue of TBPH in glia were settled at 22°C, the constitutive rescue of EAAT1 was performed at 25°C and the constitutive TBPH silencing were done at 29°C.

### Life span

Adult flies were collected for two days and divided in groups of 20 flies (1:1 female:male) into fresh tubes and transferred into new tubes every third day annotating the number of dead flies. Approximately 100 flies were tested per each genotype. The experiments for the constitutive rescue of TBPH in glia were settled at 22°C and the constitutive TBPH silencing were done at 29°C.

### TARGET system

TARGET system (temporal and regional gene expression targeting system) is a technique based on the ubiquitous expression (under tubulin promoter) of the temperature sensitive engineered yeast GAL80 protein (GAL80^TS^). GAL80^TS^ regulates GAL4 in a temperature-dependent manner in embryos, larvae, pupae and adults, showing a maximal GAL4 repression capacity at 18°C and an abolishment of its repression at 31°C (28). We generated, by standard genetic crosses, flies carrying a single copy of the tubulin-GAL80^TS^ construct, repo-GAL4 driver and UAS-transgene of interest. An age-synchronized embryo population was grown at 18°C and, same stage larvae or same age adult flies and their respective control groups were shifted at 25°C or 29°C or 31°C, in accordance to the needs of the experiment.

*Acute silencing through TBPH-RNAi expression in glia*: an age-synchronized embryo was grown at 18°C and then L3 larvae were shifted at 29°C for 24 h and then larval movement was analyzed. Similar experiment was performed in adults flies, shifting them at day 1 of adulthood to 29 and 31°C to measure life span and climbing ability.

*Late TBPH transgenic expression in glia*: age-synchronized embryos were grown at 18°C and then early L3 larvae were shifted at 25°C for 24 h and then larval movement was analyzed. Similar experiment was performed in adults flies, shifting them at day 1 of adulthood to 25°C and analyzed in climbing after 24 h.

### Drug treatment

Formula 4–24 Instant Drosophila medium (Carolina) was used for drug treatment experiment. The final concentration of NDGA was 1 mm, from 500 mm stock solution in 99.8% ethanol stored at −20°C in single aliquots. For control flies same amount of ethanol was added in the food. Parental flies were allowed to deposit embryos for 1 day and were subsequently removed; embryos were maintained at 29°C on a 12:12 h light:dark cycle and the larval movement of third instar larvae were assessed.

### Electrophysiology

Experiments were performed at 20–22°C on L3 larvae body wall preparations dissected in Ca^2+^ free HL3 saline (29) and pinned on the silicone coated surface of a 35 mm Petri dish (30). After dissection, Ca^2+^ free HL3 saline was replaced with 1 mm Ca^2+^ HL3 and body wall were left to incubate in this saline for at least 15 min before starting intracellular recordings. Electrophysiological recordings were performed on fibers 6/7 of abdominal segment 3 or 4 using intracellular glass microelectrodes 1.2 mm o.d., 0.69 mm i.d., 10–12 MOhm resistance (Science Products, Germany) filled with a 1:2 solution of 3 M KCl and 3 M CH_3_COOK. Fibers with a membrane resting potential lower than −60 mV were discarded. Two fibers for each larval body wall were utilized for electrophysiological recording. Signals were amplified in current-clamp mode by a bridge amplifier (BA-01X, NPI, Germany). Evoked neurotransmitter release was analyzed by intracellular recording EJPs. EJPs were elicited by stimulating at 0.5 Hz (stimulus duration 0.4 ms, 1.5 threshold voltage) the single segmental nerve innervating abdominal segment A3 or A4. The segmental nerve was stimulated using a suction microelectrode, filled with extracellular bathing solution and connected to a stimulator (S88, Grass, USA) via a stimulus isolation unit (SIU5, Grass, USA) in a capacitive coupling mode. Amplified signals were digitized using a A/D interface (National Instruments, USA) and then fed to a PC for both on-line visualization and off-line analysis using appropriate software (WinEDR, Strathclyde University; pClamp, Molecular Devices, USA). Statistical analysis and graph construction were carried out using Prism software (GraphPad, USA).

### Immunohistochemistry

To label the peripheral glia that covers the presynaptic terminals at the NMJ level, we expressed a membrane tethered green fluorescent protein (GFP, UAS-mCD8-GFP) in glia using the glial-specific driver *repo-*GAL4 and utilized anti-GFP antibodies to enhance the signal by immunocytochemistry. The presynaptic terminals instead were detected with antibodies against HRP that cross-react with neuron-specific membrane antigens.

Third instar larval body were dissected in saline solution (CaCl_2_ 0.1 mm, MgCl_2_ 4 mm, KCl 2 mm, NaCl 128 mm, sucrose 35.5 mm and Hepes 5 mm pH 7.2), fixed for 20 min in 4% paraformaldehyde (5 min in methanol at −20°C for anti-GluRIIA), washed in PBS 0.1% Tween 20, blocked with 5% Normal Goat Serum (Vector Laboratories) in PBS 0.1% Tween. Primary antibodies were incubated over night at 4°C and then the secondary antibodies were incubated for 2 h at room temperature. SlowFade Gold (Life Technologies) has been used for the mounting. Images were acquired on a Zeiss 510 Meta confocal microscope with a 63× oil lens and 40× lens, then analyzed using ImageJ (Wayne Rasband, NIH). Dilutions of antibodies are reported: anti-HRP (Jackson 1:150), anti-GFP (Life Technologies 1:200), anti-Syx 8C3s (DSHB 1:15), anti-Dlg 4F3c (DSHB 1:250), anti-GluRIIA 8B4D2 (DSHB 1:15), anti-HRP-Cy3 (Jackson 1:150), Alexa-Fluor® 488 (mouse, rabbit 1:500), Alexa-Fluor® 555 (mouse, rabbit 1:500).

### Quantification of confocal images

Animals analysed for these experiments were processed simultaneously and images acquired using the same microscope settings. The NMJs of second abdominal segment on muscles 6 and 7 were analyzed. Images were processed with ImageJ and then statistically analyzed using Prism (GraphPad, USA). For the pre and postsynaptic marker quantification the samples were double labeled with anti-HRP and the marker of interest: the mean intensity of both the marker and the HRP was quantified and a ratio calculated, adapted from (31). The quantification of glial area was calculated analysing the ratio between the area occupied by glial tissue versus the area of the presynaptic terminal. The quantification of boutons shape was done considering as regular the boutons with equal diameter on both axis and as irregular the boutons with deformed and fusiform shape (different axis length).

### Western blot

Proteins were extracted from adult heads or from dissected larval brains. The material has been squeezed in lysis buffer 1 X (Lysis buffer composition 1.5 X: 225 mm NaCl, 15 mm Tris, 7.5 mm EDTA, 15% glycerol, 7.5 mm EGTA, 75 mm NaF, 6 M urea, 7.5 mm DTT and protease inhibitor) and then clarified by a short centrifugation at 0.5 × *g*. The proteins were separated by SDS-PAGE and blotted on 0.2 µm nitrocellulose membrane (Whatman Protran). Membranes were blocked overnight in 5% nonfat dried milk in TBS-0.01% Tween 20 and probed with primary antibodies: anti-TBPH (in house 1:2000) and anti-Tubulin (Calbiochem 1:4000). Goat anti-rabbit and goat anti-mouse IgG HRP conjugated were used as secondary antibodies (Thermo Scientific 1:1000). Detection was done with Femto SuperSignal substrate (Thermo Scientific). Band intensity was quantified using ImageJ and normalized on loading control. In every lane the mean percentage obtained out of at least three experiments has been shown and normalized to control genotype.

### Statistical analysis

All statistical analysis was performed with Prism (GraphPad, USA) version 6.0. Student's T test was used to compare two different groups and one-way ANOVA was used to test more than two groups, applying the Bonferroni correction as post multiple comparison test. In all figures a graph reporting the mean value and the standard error of the mean (SEM) was reported. Log-rank test was used to compare survival curves of different genotypes.

## Supplementary Material

Supplementary Material is available at *HMG* online.

## Funding

This work was supported by AriSLA (CHRONOS). Funding to pay the Open Access publication charges for this article was provided by AriSLA (CHRONOS).

## Supplementary Material

Supplementary Data
