# Supplementary material for: Glial TDP-43 regulates axon wrapping, GluRIIA clustering and fly motility by autonomous and non-autonomous mechanisms
Source: Hum Mol Genet. 2015 Aug 13;24(21):6134–45. doi: 10.1093/hmg/ddv330 (PMC4599672; doi:10.1093/hmg/ddv330)
Supplement: Supplementary Data [file supp_24_21_6134__index.html]

Glial TDP-43 regulates axon wrapping, GluRIIA clustering and fly motility by autonomous and non-autonomous mechanisms — Glial TDP-43 regulates axon wrapping, GluRIIA clustering and fly motility by autonomous and non-autonomous mechanisms — Supplementary Data 

# Glial TDP-43 regulates axon wrapping, GluRIIA clustering and fly motility by autonomous and non-autonomous mechanisms

## Supplementary Data

Supplementary Data

- Supplementary Data - Docx file
