# Supplementary material for: Glial TDP-43 regulates axon wrapping, GluRIIA clustering and fly motility by autonomous and non-autonomous mechanisms
Source: Hum Mol Genet. 2015 Aug 13;24(21):6134–45. doi: 10.1093/hmg/ddv330 (PMC4599672; doi:10.1093/hmg/ddv330)
Supplement: Supplementary Data [file supp_ddv330_ddv330supp.docx]

**SUPPLEMENTARY MATERIAL**

**Glial TDP-43 Regulates Axon Wrapping, GluRIIA Clustering and Fly Motility by Autonomous and Non-Autonomous Mechanisms**

Giulia Romano^1,2^, Chiara Appocher^1,2^, Michele Scorzeto^3^, Raffaella Klima^2^, Francisco E. Baralle^2^, Aram Megighian^3^ and Fabian Feiguin^2*^

^2^International Centre for Genetic Engineering and Biotechnology, Padriciano 99, 34149 Trieste, Italy. ^3^Department of Biomedical Sciences, University of Padova, via Marzolo 3, 35131 Padova, Italy.

^1^ Equally contributed authors.

^*^ Correspondence to: [fabian.feiguin@icgeb.org](mailto:fabian.feiguin@icgeb.org)

**Supplementary Material and Methods**

**FM 1-43 *in vivo* assay**

FM 1-43 dye labeling, unloading and quantification of intensities was performed as described in (1). FM 1-43 dye uptake was performed for 1 minute of 90 mM KCl stimulation and then the unloading was performed for 7 minutes of KCl stimulation. Images were captured with a Nikon DS-Qi2 microscope and 60x /1.0W lens.

**Brains Dissection**

Third instar larval brains were dissected in PB 0.3% Triton X-100 and fixed in 4% paraformaldehyde for 20 minutes. Larval brains have been washed in PB 0.3% Triton X-100, blocked with 5% Normal Goat Serum, incubated over night at 4°C with primary antibodies and then further incubated for 2 hours at room temperature with the secondary antibodies. SlowFade Gold has been used for the mounting. Images were acquired on a Zeiss 510 Meta confocal microscope with a 63x oil lens and 40x lens, then analyzed using ImageJ (Wayne Rasband, NIH). Dilutions of antibodies are reported: anti-TBPH (in house 1:200), anti-Repo 8D12 (DSHB 1:200), anti-elav (DSHB 1:250), Alexa-Fluor® 488 (mouse 1:500), Alexa-Fluor® 555 (rat, rabbit 1:500).

**
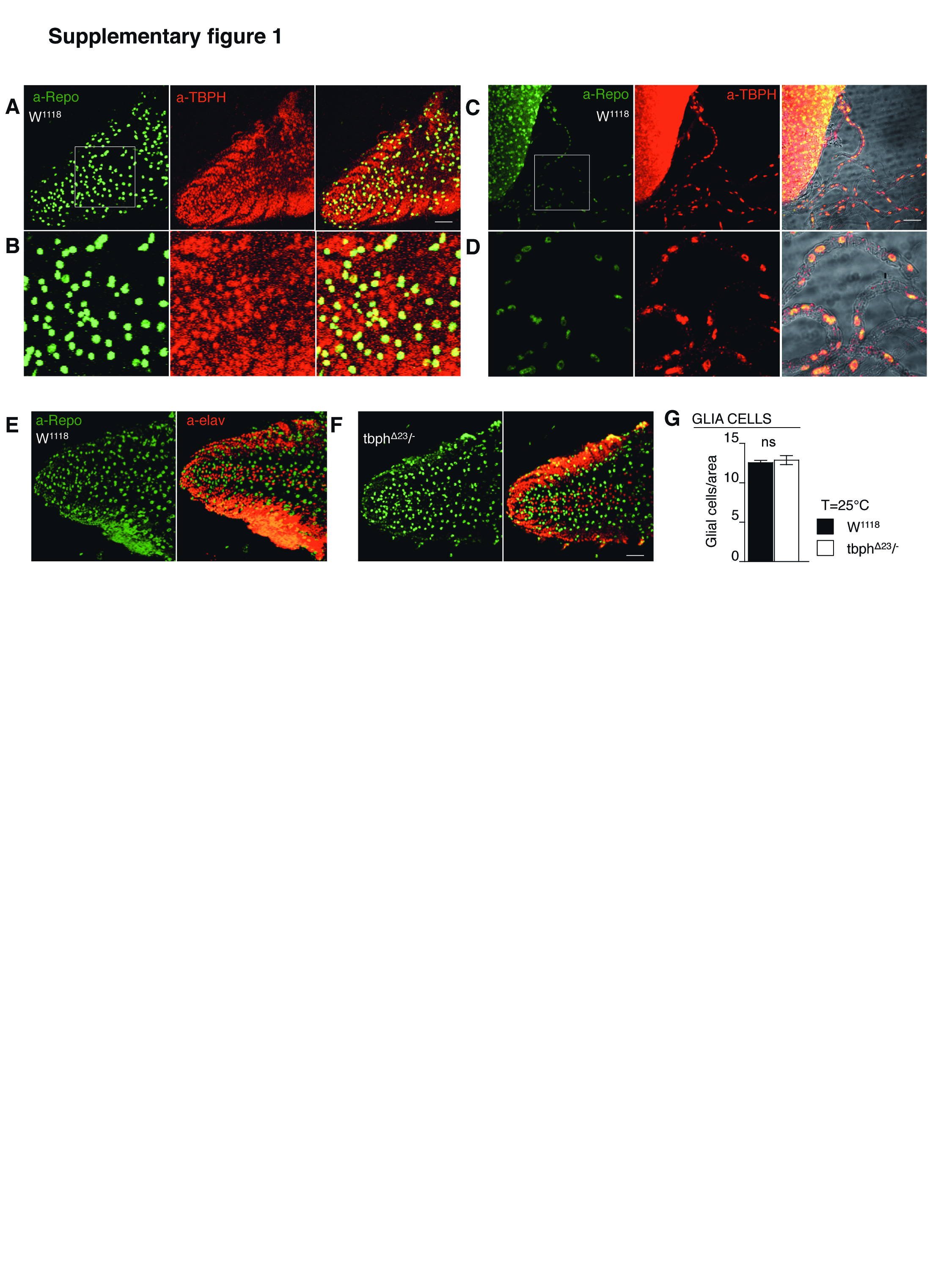
**

**Supplementary figure 1.** TBPH distribution in glial cells and quantification of glial cells in TBPH minus larvae. **(A,B)** Confocal images stained with anti-TBPH (in red) and anti-Repo (in green) of **(A)** L3 ventral ganglion and (**B**) its detail at higher magnifications (2x) in W^1118^. (**C**) Fluorescence and bright field images of larval nerves covered by glial cells stained with anti-Repo (in green) and anti-TBPH (in red) in W^1118^, in (**D**) its detail at higher magnifications (2x). (**E,F)** Confocal images stained with anti-Repo (in green) and anti-elav (in red) from (**E**) W^1118^ and (**F**) *tbph^∆23/-^* L3 ventral ganglion. **(G)** The number of Repo positive cells was counted in a fixed area (segment A5, A6, and A7) of L3 ventral ganglion considering only the glial cells clustered in the midline. n=15 (Scale bar 20 µm).

**
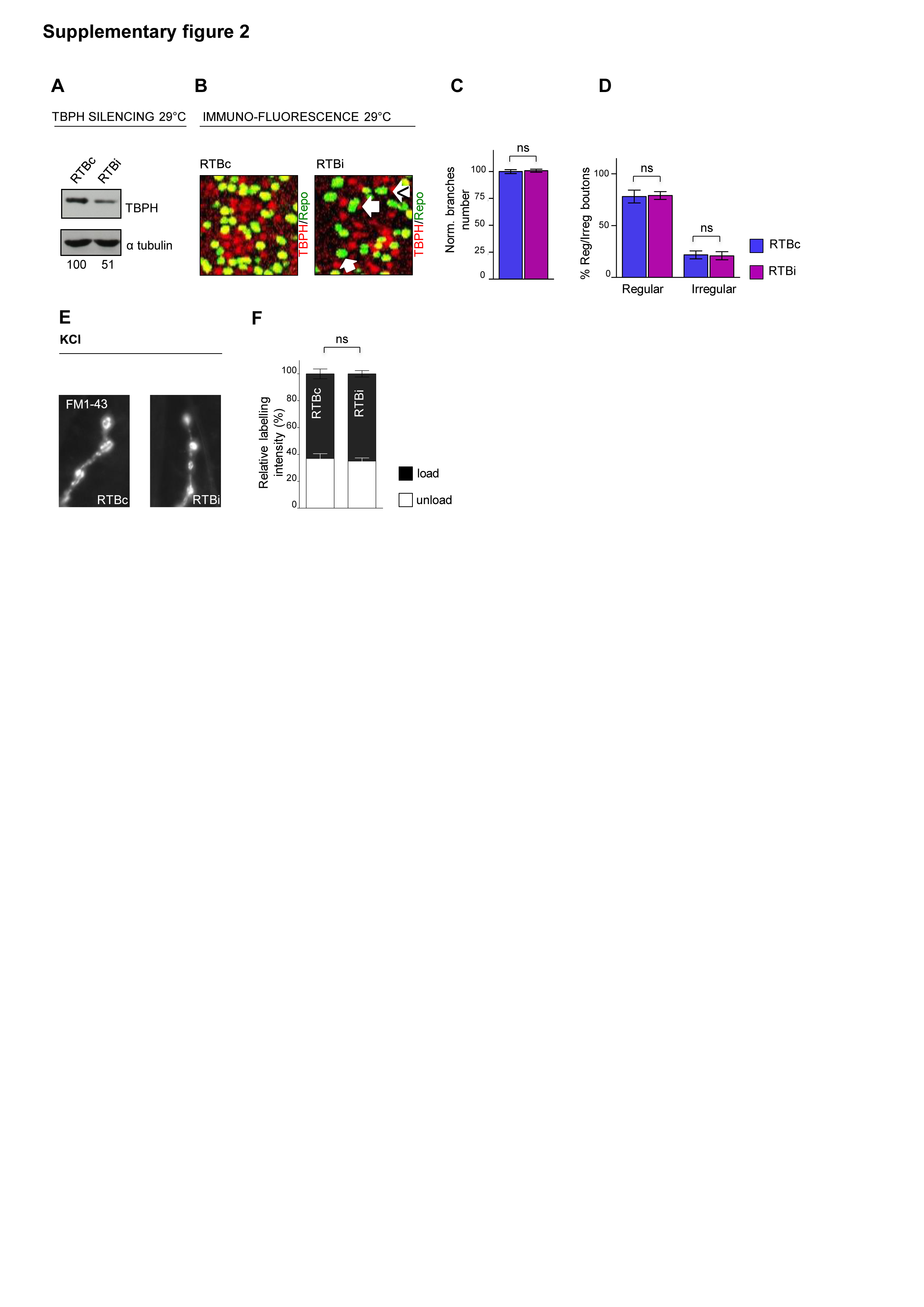
**

**Supplementary figure 2.** Constitutive silencing of TBPH in glia. (**A**) Western blot of L3 brains probed with anti-TBPH and alpha-tubulin in RTBc (UAS-Dcr2;*tbph^∆23^*/+;*repo*-GAL4,UAS-GFP/+) and RTBi (UAS-Dcr2;*tbph^∆23^*/+;*repo*-GAL4,UAS-GFP/TBPH-RNAi). Quantification of normalized protein amount was reported below each lane, n=3. (**B**) Confocal images of larval brains stained with anti-TBPH (in red) and anti-Repo (in green) in RTBc and RTBi. White arrows show the reduced amount of TBPH protein in glial nuclei of RTBi larvae. The level of TBPH was reduced of 45% in glia nuclei compare to the control. Number of glial nuclei quantified n=100. (**C**) Quantification of the number of branches in muscle 6/7 in RTBc and RTBi larvae. n=15 (**D**) Quantification of regular and irregular boutons present in muscle 6/7 in RTBc and RTBi larvae, n=15. (**E)** FM 1-43 dye uptake after 1 minute of 90 mM KCl stimulation in RTBc and RTBi larvae. (**F**) Quantification of labeling bouton showed the loading and unloading of FM 1-43 dye in TRBc and TRBi. The unload was evaluated after 7 minutes of a second KCl stimulation, n=3 larvae. ns=not significant, calculated by T-test. Error bars SEM.

**
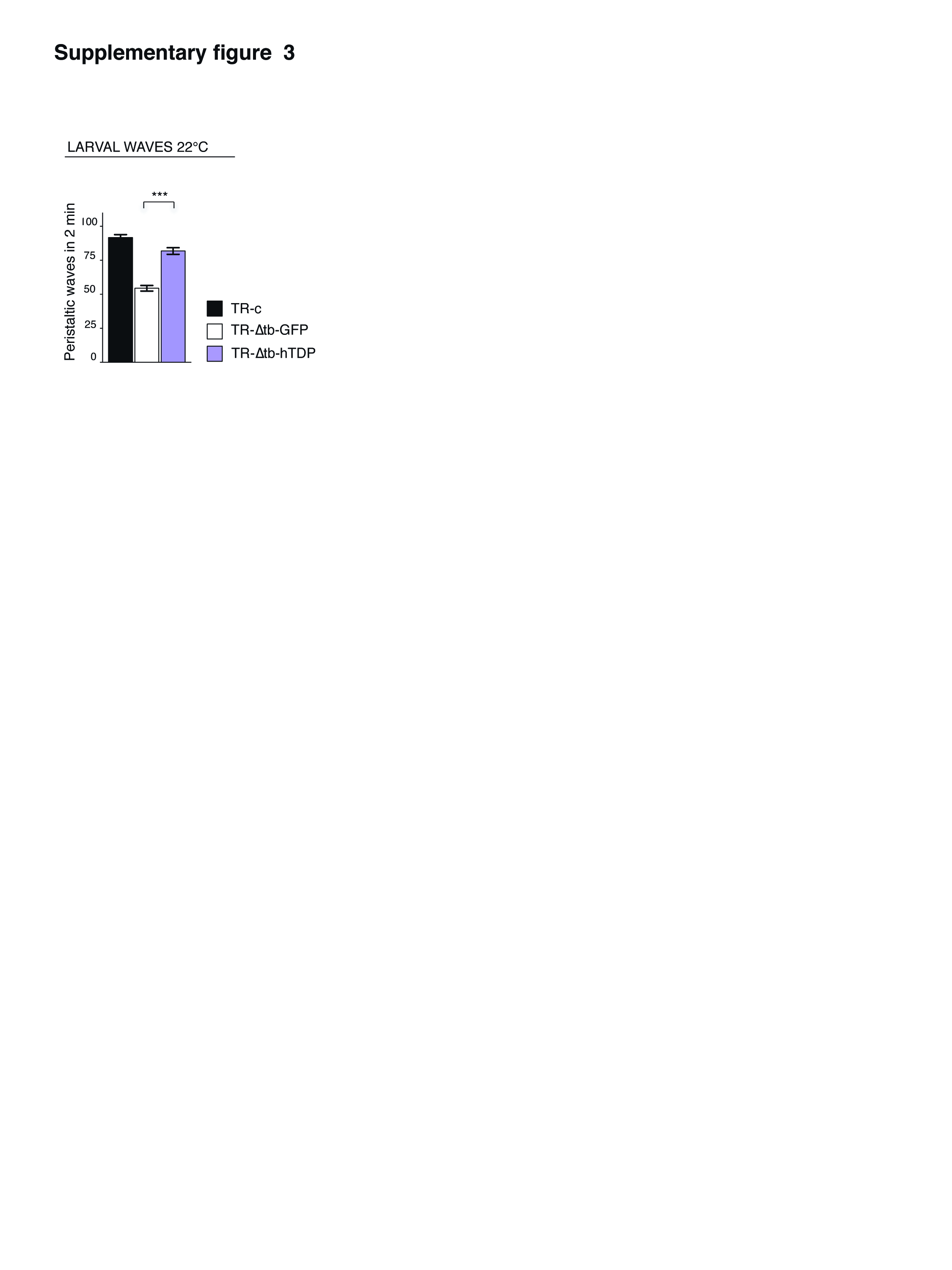
**

**Supplementary figure 3.** Expression of hTDP-43 in glia recovers larval motility. Number of peristaltic waves in TR-c (*tubulin*-GAL80^TS^,*tbph^∆23^*/+;*repo*-GAL4,UAS-GFP/+), TR-∆tb-GFP (*tubulin*-GAL80^TS^,*tbph^∆23^*/ *tbph^∆23^*;*repo*-GAL4,UAS-GFP/+) and TR-∆tb-hTDP (*tubulin*-GAL80^TS^,tbph^∆23^*/tbph^∆23^*;*repo*-GAL4,UAS-GFP/UAS-hTDP-43) larvae, n=20. ***p<0.001 calculated by one-way ANOVA. Error bars SEM.

**
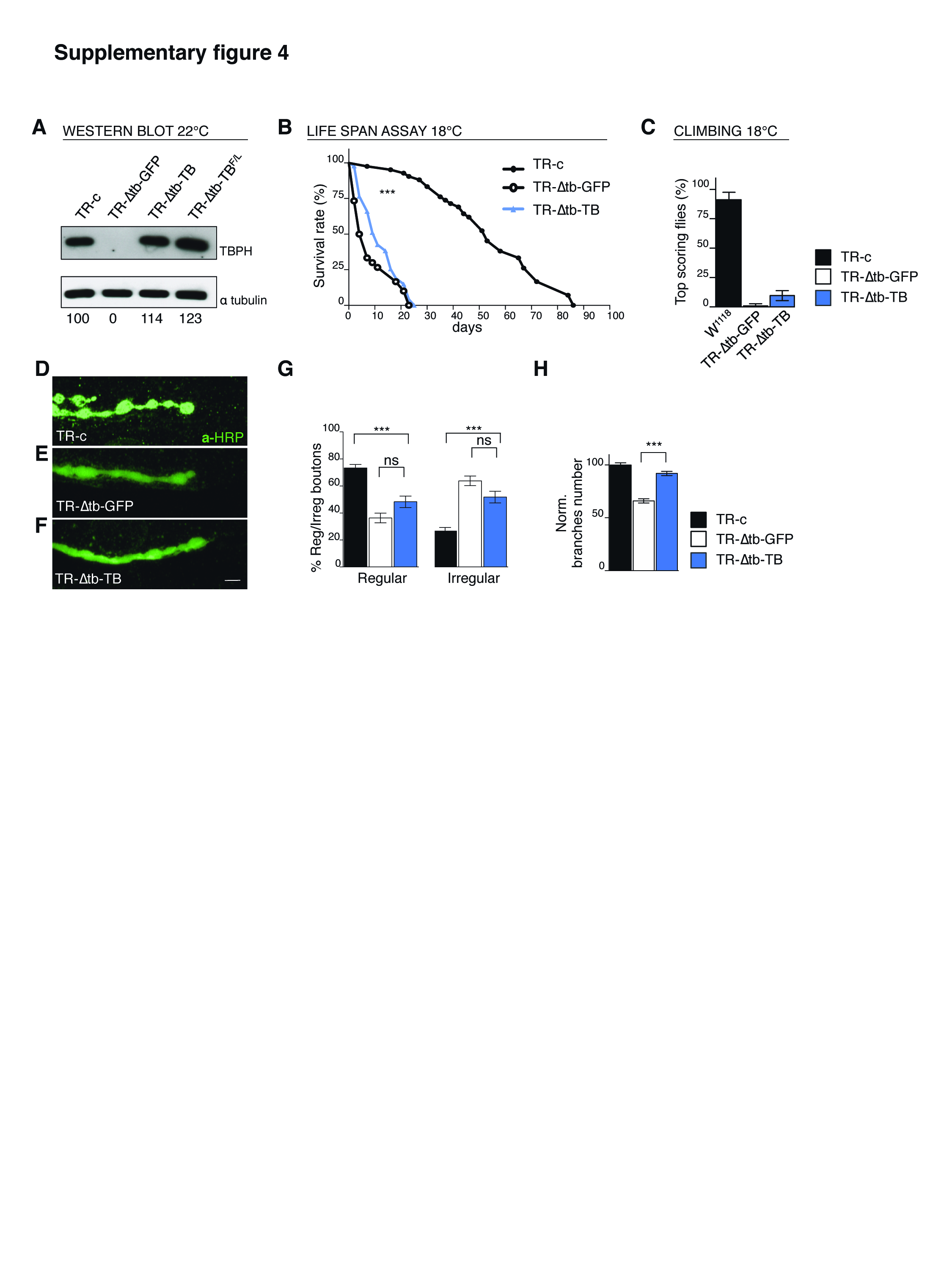
**

**Supplementary figure 4.** Expression of transgenic TBPH in glial tissue of TBPH mutant. (**A**) Western blot analysis of third instar larval brains probed with anti-TBPH and alpha-tubulin in TRc (*tubulin*-GAL80^TS^,*tbph^∆23^*/+;*repo*-GAL4,UAS-GFP/+), TR-∆tb-GFP (*tubulin*-GAL80^TS^,*tbph^∆23^*/*tbph^∆23^*;*repo*-GAL4,UAS-GFP/+), TR-∆tb-TB (*tubulin*-GAL80^TS^,tbph^∆23^*/tbph^∆23^*,UAS-TBPH;*repo*-GAL4,UAS-GFP/+) and TR-∆tb-TB^F/L^ (*tubulin*-GAL80^TS^,*tbph^∆23^*/*tbph^∆23^*;*repo*-GAL4,UAS-GFP/UAS-TBPH^F/L^). Quantification of normalized protein amount was reported below each lane, n=3. (**B**) Life span performed in TR-c TR-∆tb-GFP and TR-∆tb-TB adult flies, maintained at restrictive temperature (18°C). (**C**) Climbing ability of TR-c, TR-∆tb-GFP and TR-∆tb-TB adult flies maintained at restrictive temperature (18°C). (**D-F**) Confocal images of NMJ terminals labeled with anti-HRP in TR-c, TR-∆tb-GFP and TR-∆tb-TB larvae, n=15. (**G**) Quantification of regular and irregular boutons present in muscle 6/7, n=15. **(H)** Quantification of the number of branches in muscles 6/7 in TR-c, TR-∆tb-GFP and TR-∆tb-TB ***p<0.001 calculated by one-way ANOVA. Error bars SEM.

**
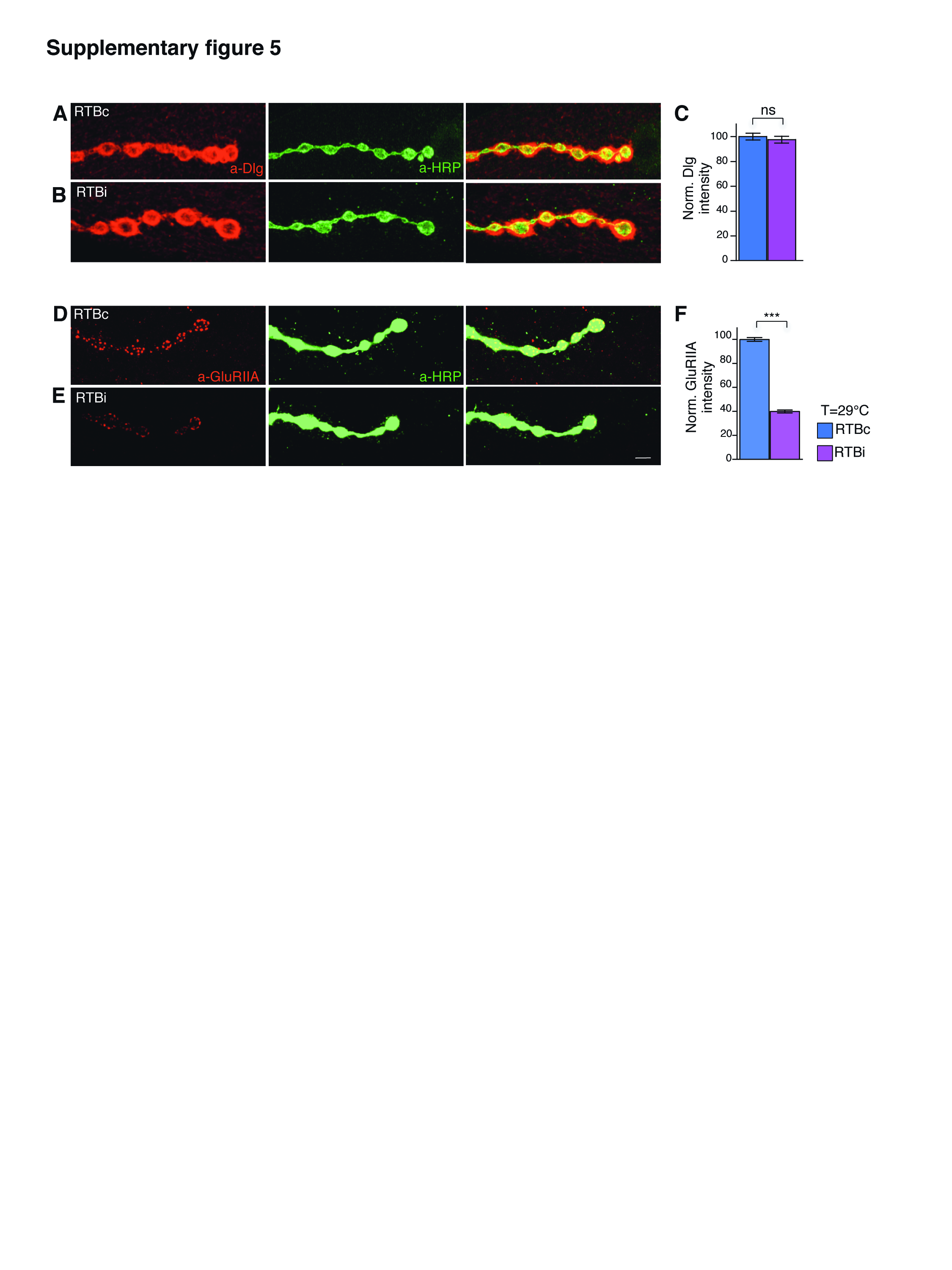
**

**Supplementary figure 5.** Constitutive silencing of TBPH in glia. (**A,B**) Confocal images of L3 NMJ terminals in muscle 6/7 stained with anti-HRP (in green) and anti-Dlg (in red) in (**A**) RTBc **(**UAS-Dcr2;*tbph^∆23^*/+;*repo*-GAL4,UAS-GFP/+**)** and in (**B**) RTBi (UAS-Dcr2;*tbph^∆23^*/+;*repo*-GAL4,UAS-GFP/TBPH-RNAi). (**C**) Quantification of Dlg intensity, n=250 boutons (**D,E**) Confocal images of L3 NMJs presynaptic boutons in muscle 6/7 stained with anti-HRP (in green) and anti-GluRIIA (in red) in (**D**) RTBc and in (**E**) RTBi. (**F**) Quantification of GluRIIA intensity, n=250 boutons. Scale bar 5 µm. ns= not significant, ***p<0.001 calculated by T-test. Error bars SEM.

**
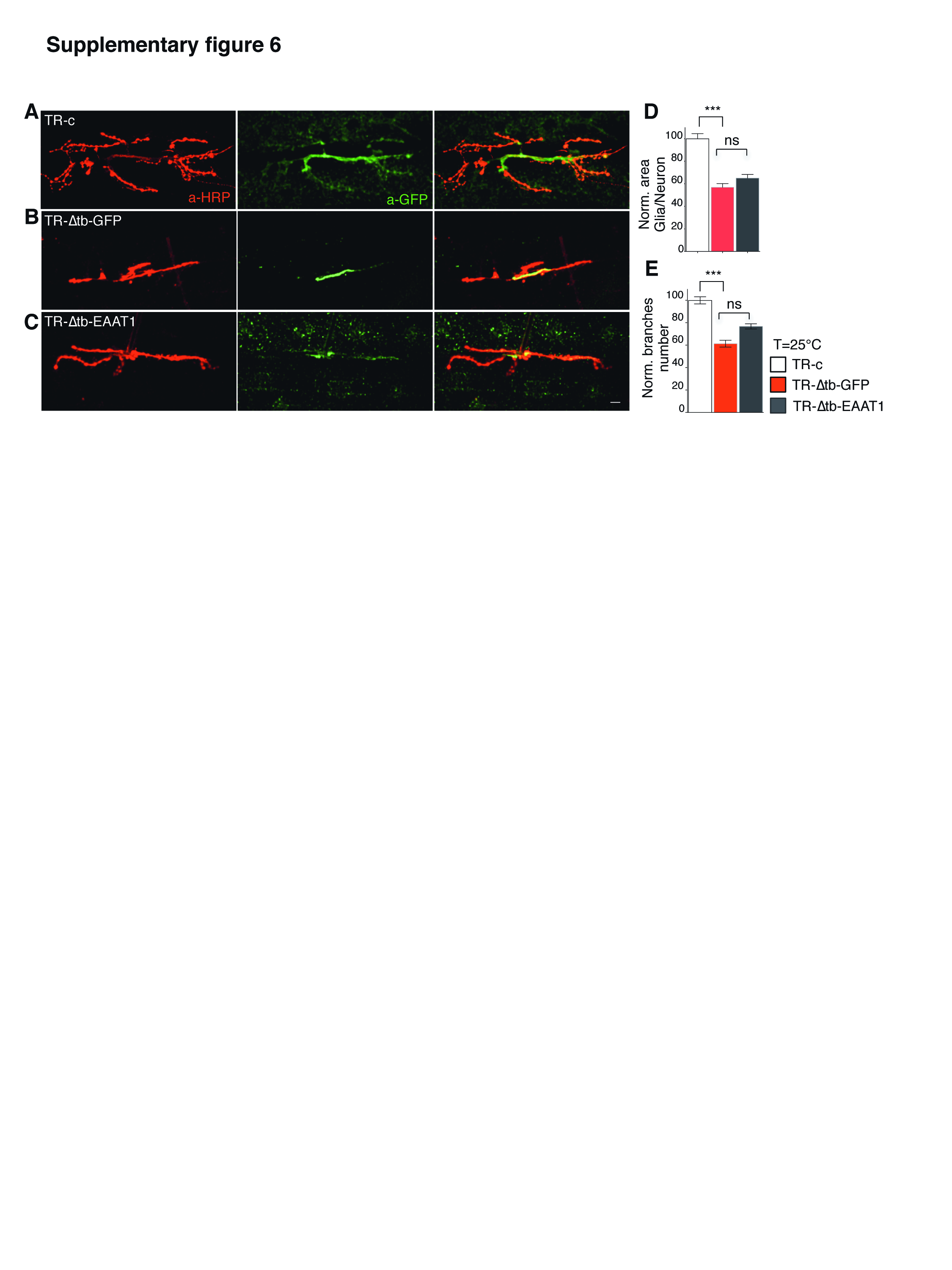
**

**Supplementary figure 6.** Expression of EAAT1 transgene in TBPH mutant larvae. (**A-C**) Confocal images of third instar NMJs expressing mCD8-GFP in glial cells using *repo*-GAL4 double labeled with anti-HRP (in red) and anti-GFP (in green) in (**A**) TR-c (*tubulin*-GAL80^TS^,tbph^∆23^*/+*;*repo*-GAL4,UAS-GFP/+), in (**B**) TR-∆tb-GFP (*tubulin*-GAL80^TS^,tbph^∆23^*/tbph^∆23^;repo-*GAL4,UAS-GFP*/+*) and in (**C**) TR-∆tb-EAAT1 (*tubulin*-GAL80^TS^,tbph^∆23^*/ tbph^∆23^; repo-*GAL4,UAS-GFP/UAS-dEAAT1). (**D**) Quantification of glial area, n=15. (**E**) Quantification of the number of branches in muscle 6/7, n=15. ns=not significant, ***p<0.001 calculated by one-way ANOVA. Error bars SEM.

**REFERENCES**

1. Verstreken,P., Ohyama,T. and Bellen,H.J. (2008) FM 1-43 labeling of synaptic vesicle pools at the Drosophila neuromuscular junction. *Methods Mol. Biol. Clifton NJ*, **440**, 349–369.
